# Highlight—Evolution on the Outskirts: Virtual Issue on Viral Evolution

**DOI:** 10.1093/gbe/evab027

**Published:** 2021-03-05

**Authors:** Casey McGrath

Despite the fact that viruses are among the simplest biological entities—consisting only of DNA or RNA encapsulated in a protein shell—they can have devastating consequences, with viruses such as influenza, human immunodeficiency virus (HIV), and Ebola having dramatically affected the course of human history. Because they generally lack the cellular machinery necessary to reproduce, they propagate by hijacking host cells, often to the host’s detriment. Although their status as a “living” organism may be in question, there is no doubt that viruses are shaped by evolutionary forces that influence their genomes, as well as their replication, host range, virulence, and other features. With the emergence of SARS-CoV-2, a virus that has intimately affected virtually all aspects of human life for the past year, the study of viral evolution may seem more profound and relevant than ever before. *Genome Biology and Evolution’*s latest virtual issue is a collection of thought-provoking articles in the field of viral evolution from the past 2 years, providing new insight into the evolutionary mechanisms that influence viruses, their genomes, and their hosts, as well as showcasing their use in the study of evolution.

The rapid and variable mutation rates observed among viruses create challenges for studies of viral genome evolution. A study by González et al. (2019) sought to investigate the balance between beneficial and deleterious mutations by creating a mutant library of the tobacco etch potyvirus containing every possible single-nucleotide substitution of the *6K2* gene and then infecting tobacco plants with the library. By assessing the mutants remaining after competition within the host, the study revealed that most observed mutations were associated with higher fitness and the development of milder symptoms, suggesting a potential tradeoff between within-host accumulation and symptom severity in nature. A study by Willemsen et al. (2019) investigated the mutational origin and history of the papillomavirus *E5* gene, which is known to be associated with cancer development in the host. Surprisingly, the authors found that a long stretch of noncoding DNA arose in the present location of the *E5* gene at least four times during the evolution of papillomaviruses, evolving into at least three different open reading frames encoding novel proteins that are associated with distinct infection phenotypes (cutaneous warts, genital warts, or anogenital cancers). This provides a mechanism for genetic novelties in viruses and demonstrates their potential phenotypic effects. Lastly, in their paper, Ou et al. (2020) posit a theory that the metabolic background of RNA viruses—specifically the high demand for cytidine triphosphate (CTP)—results in a mutational bias toward the replacement of cytosine with uracil, which may restrict the evolutionary trajectories of these viruses.

Because viruses use the host’s machinery for their replication, they are constrained in their codon usage. Thus, another intriguing area of research involves comparing patterns of codon usage among viral genes and between viral and host genomes. Interestingly, the human hepatitis A virus (HAV) exhibits a unique codon composition, using codons that appear at intermediate frequency in the host, rather than those that are rare or abundant in the host. To understand this unique feature, D’Andrea et al. (2019) performed an experiment in which they followed the evolution of HAV under artificially induced transcription shutoff, which alters the tRNA pools available for the virus. According to the resulting genotype–phenotype maps, codon composition influenced the translational robustness and evolvability of HAV, with adaptation to transcription shutoff involving mutations that significantly impacted translation efficiency. In order to gain a better understanding of what codons are desirable in what contexts and how combinations of synonymous mutations affect viral growth, Van Leuven et al. (2021) conducted a study in which they systematically recoded bacteriophage ΦX174 genes with codons that were rarely used in its *E. coli* host. The researchers found that although additional deoptimizing mutations resulted in decreases in viral fitness, the effects were not always linear or consistent across genes, emphasizing the need for further studies to reveal how selection influences viral codon usage.

Another field of study involves understanding the effects that viruses have on their host organisms. Fiedoruk et al. (2020) performed an analysis of the filamentous bacteriophages of *Pseudomonas aeruginosa*, which are known to contribute to the bacterium’s pathogenicity by promoting biofilm formation, suppressing phagocytosis by macrophages, and sequestering cationic antibacterial compounds. The study revealed that there are actually two distinct evolutionary lineages of these phages, each with unique structural properties that may have differential impacts on *P. aeruginosa* virulence and antimicrobial resistance. A study by Simons et al. (2019) investigated the effects of simian immunodeficiency virus (SIV) in the Ugandan red colobus and found no evidence for chronic immune activation, suggesting that, unlike HIV in humans, SIV is not immunocompromising in these primates.

The effects of a virus can also persist long after a viral infection has been cleared. For example, viruses can leave traces in the host genome in the form of endogenous viral elements. Cheng et al. (2020) analyzed the genomes of insects and other arthropods and found evidence for nudivirus-like genes in dozens of different taxa not previously known to be hosts to this viral lineage, greatly extending the host range of nudiviruses. Gene transfer agents (GTAs) offer another example of the lasting influence of viruses on hosts. These sequences are so named because they enable the exchange of genetic material between bacteria, although they are thought to derive originally from viruses. In fact, according to a study by Kogay et al. (2019), they are often misannotated as viral proteins in genetic databases. Kogay et al. developed a machine-learning algorithm to distinguish true GTAs from their viral homologs based on differences in the amino acid composition between the two types of protein. Using this tool, they identified GTAs in over half of the alphaproteobacterial genomes examined, broadening the known distribution of these sequences.

Together, these studies provide a deeper understanding of not only viruses themselves but also the potentially lasting effects on their hosts, which include virtually all cellular organisms across the tree of life. *Genome Biology and Evolution* continues to provide a platform for disseminating new insights and discussing new methods of investigation into outstanding questions in the field of viral evolution.
